# Screen-Printed Corrosion-Resistant and Long-Term Stable Stretchable Electronics Based on AgAu Microflake Conductors

**DOI:** 10.1021/acsami.2c22199

**Published:** 2023-02-23

**Authors:** Ulrika Boda, Jan Strandberg, Jens Eriksson, Xianjie Liu, Valerio Beni, Klas Tybrandt

**Affiliations:** † Bio and Organic Electronics Unit, Department of Smart Hardware, Digital Systems Division, RISE Research Institutes of Sweden AB, 602 21 Norrköping, Sweden; ‡ Department of Physics, Chemistry and Biology, Linköping University, 581 83 Linköping, Sweden; § Laboratory of Organic Electronics, Department of Science and Technology, Linköping University, 602 21 Norrköping, Sweden

**Keywords:** stretchable electronics, soft electronics, printed electronics, gold, silver flakes, corrosion, stability, NFC

## Abstract

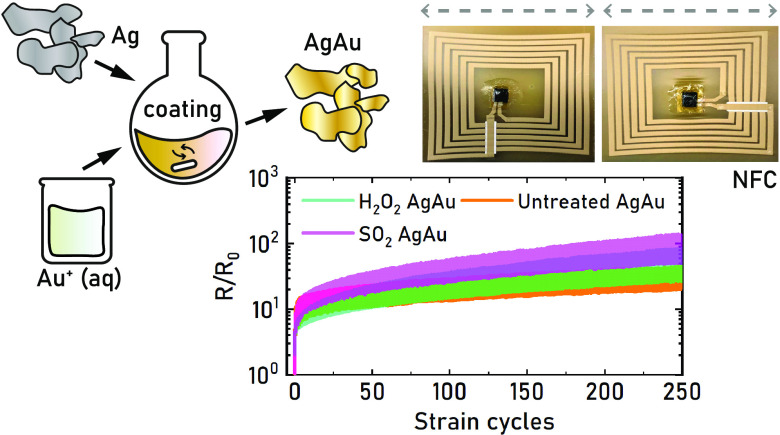

High-throughput production methods such as screen printing can bring stretchable electronics out of the lab into the market. Most stretchable conductor inks for screen printing are based on silver nanoparticles or flakes due to their favorable performance-to-cost ratio, but silver is prone to tarnishing and corrosion, thereby limiting the stability of such conductors. Here, we report on a cost-efficient and scalable approach to resolve this issue by developing screen printable inks based on silver flakes chemically coated by a thin layer of gold. The printed stretchable AgAu conductors reach a conductivity of 8500 S cm^–1^, remain conductive up to 250% strain, show excellent corrosion and tarnishing stability, and are used to demonstrate wearable LED and NFC circuits. The reported approach is attractive for smart clothing, as the long-term functionality of such devices is expected in a variety of environments.

## Introduction

Recent interest in neural interfacing, wearable technology, and e-skin has driven the development of electronics from traditional rigid or flexible form factors to soft and elastic devices.^1−5^ For skin patches and wearable technology, strain up to 30% should be accommodated by conductors without significant deterioration in electrical performance,^6^ a value which can rise to above 100% for application at joints on the human body or for e-skin on robots with mobility.^7^ Stretchable conductors can be achieved by macroscale patterning of metal films into serpentine or kirigami geometries.^8−10^ Conventional microfabrication strategies can be employed for such conductors, including processing steps like thermal evaporation and sputtering, photolithography, and dry and wet etching. The environmental footprint and limited throughput of such methods have generated interest in alternative material-based approaches that can be compatible with high-throughput methods like printing. As a result, a wide range of intrinsically stretchable conductors have been developed, which can retain a sufficiently low resistance during repeated mechanical stretching in one or several directions.^11−16^ A common approach for manufacturing stretchable conductors is to mix conductive metallic fillers with insulating elastomers to create composite systems that are potentially scalable and printable.^17−21^ Screen printing is a very attractive method since it is cost-efficient, environmentally friendly (low material waste and often based on low-toxic substances), and suitable for large-scale production on a variety of flexible and stretchable substrates. Screen printing can produce any two-dimensional (2D) pattern with resolutions down to 100 μm readily achievable,^22^ while resolutions down to 30–40 μm have been reported when specialized equipment, inks, and substrate parameters are adopted.^23^ As the processing parameters of the ink can often be kept below 130 °C, soft and sensitive substrates can be used.^24^ Silicone elastomers, such as PDMS, are often used in stretchable electronics due to their good mechanical properties and chemical stability.^19,25,26^ However, their low surface energy and adhesion, together with contamination issues, make them less suitable for screen printing.^27,28^ Instead, some of the most commonly and successfully employed elastic substrates for screen printing are polyurethanes (PU) and thermoplastic polyurethane variants (TPU) due to their good elasticity, stability, and higher surface energy, which facilitates additive manufacturing.^27,29^ The properties of the elastic matrix of a stretchable screen printing ink are of equal importance, as the ink needs similarly high surface energy as the substrate to adhere well and avoid delamination and cracking during deformation. State-of-the-art stretchable conductive inks commonly consist of silver (Ag) or carbon filler particles and an elastomer binder, together forming a solid yet soft stretchable composite conductor after printing and curing steps.^29−32^ Carbon materials, such as carbon black, graphite and graphene nanoplatelets, and carbon nanotubes, generally lack the high conductivity necessary for interconnects in many applications.^33−35^ Silver is the most widely used conductive filler in screen printing inks, being relatively affordable and highly conductive. Silver nanowires are a popular filler choice in stretchable conductive composites due to their high aspect ratio and low percolation threshold;^31^ however, their relatively high cost has limited their use in screen printing inks. Instead, silver flakes have gained popularity in commercial screen printing inks, as they combine favorable aspect ratio with low price.^31,36^ Unfortunately, despite often being classified as a noble metal, Ag lacks long-term stability to corrosion and tarnishing when exposed to moisture and common air contaminants, such as sulfuric compounds.^37,38^ Moreover, Ag ions can migrate from nanosilver during oxidation of the surface and potentially be harmful in biological environments.^39^ This can be problematic for wearable applications in clothing and implants, as the printed devices are expected to last for a long time. This is especially challenging in high-performance applications like near-field communication (NFC) technology, which has attracted growing interest in the areas of IoT and wearables lately. NFC technologies allow for short-distance two-way communication and energy transfer between electronic devices such as mobile phones and wearable sensors,^40^ making the technology attractive for flexible and stretchable wearable/implantable bioelectronics.^41^ A major challenge for stretchable printed NFC solutions is the high conductivity requirements on the inks, which becomes even more problematic if the printed conductors corrode and tarnish with time.

Gold (Au) is a corrosion and tarnishing-resistant alternative to silver,^42^ but it is too expensive to be used in many printed electronics applications. A compromise is to chemically coat silver particles with a thin layer of protective gold, which successfully has been achieved for silver nanowires and nanoparticles to prevent corrosion.^43−46^ However, to our knowledge, there have been no previous reports on long-term stable stretchable inks and conductors based on such an approach.

Here, we present the first corrosion-resistant fully screen-printed metallic stretchable conductors based on Au-coated Ag microflakes (AgAu flakes). A large-scale aqueous coating process was developed based on the non-toxic electroless deposition of Au nanolayers onto microscale Ag flakes, and screen printing inks were formulated based on biocompatible water-dispersed polyurethane and harmless solvents. The printed AgAu conductors showed good conductivity and stability in corrosive and tarnishing environments where Ag flake conductors were severely affected. To demonstrate the capability of the developed ink, stretchable LED circuits and NFC skin patches were fabricated and evaluated under strain.

## Materials and Methods

### Samples and Statistics

Wherever average numbers are reported, the number of measurements given as *n* = *x* refers to the number of individual samples that were measured unless stated otherwise. Standard deviation reported as ± values refers to the sample population.

### Chemicals and Materials

2-Hydroxyethyl cellulose, NaOH, Na_2_SO_3_, poly(vinylpyrrolidone) (*M*_w_ ∼ 55 k), and (+)-sodium l-ascorbate were purchased from Merck. HAuCl_4_ was purchased from ChemPUR GmbH. Kronos TiO_2_ 2190 powder was purchased from Omya AB. Water-borne PU dispersion Baymedix CD102 as well as 50 μm thick thermoplastic polyurethane ether film substrate Platilon U073 (Shore A 87) with a polyethylene carrier foil attached were both obtained from Covestro AG. NHS3100 chips were produced by NXP Semiconductors. Z-conductive Tape 9703 and Tegaderm transparent film roll were purchased from 3M. Commercial flexible Ag ink CI-1036 was purchased from Engineered Materials Systems, Inc. (EMS).

### Au Aging

A total of 3.88 mL of HAuCl_4_ (252 mM in DI water) was combined with 38.53 mL of DI water and 6.06 mL of NaOH (1 M in DI water) and stirred on a hotplate at the lowest possible speed for 15 min at 60 °C. The resulting pale green/yellow solution was cooled for 5 min under cold running tap water. Then, 29.11 mL of Na_2_SO_3_ (0.1 M in DI water) was added and gently swirled, and the obtained solution was aged undisturbed for 24 h in the dark.

### Ag Flake Cleaning

A total of 1.5 g of commercial Ag flakes (10 μm, ≥99.9% trace metals basis, Merck) were rinsed with a solution consisting of 400 μL of acetic acid (80% in water), 10 mL of PVP (25 wt % solution in 99% EtOH), and an additional 29.6 mL of EtOH (99%). The obtained suspension was vortexed for 30 s at 2500 rpm and then left to stand for 7 h to remove surface fatty acids and sediment. Following sedimentation, the supernatant was removed, and the cleaned Ag flakes were rinsed with 40 mL of EtOH (99%) and left to sediment again. The supernatant was removed, and the rinsing and sedimentation steps were repeated once. If the cleaned Ag flakes were intended to be analyzed in SEM, they were subsequently rinsed and sedimented in DI water twice before analysis. If the cleaned Ag flakes were intended to be coated with Au, they were kept in EtOH solution to ensure the least amount of agglomeration prior to mixing into the reaction solution, but the EtOH supernatant was carefully removed just before the Au coating reaction ingredients were added.

### Au Coating on Ag Flakes

A pH 10.4 buffer solution was made, consisting of 11.25 mL of glycine (0.2 M in DI water), 8.685 mL of NaOH (0.2 M in DI water), and an additional 25.07 mL of DI water. To 1.5 g of cleaned Ag flakes in a 500 mL round flask were added in order: 56.60 mL of PVP (25 wt % in DI water), an additional 15 mL of DI water, 45 mL of NaOH–glycine buffer (10.4 pH), 1.94 mL of Na_2_SO_3_ (0.1 M in DI water), and 3.88 mL of sodium ascorbate (1 M in DI water). The resulting dispersion was stirred at ca 1000 rpm at room temperature, ca 22 °C. Then, 77.582 mL of the previously aged Au solution was added via rapid pipetting in portions of 10 mL at a time during stirring, after which the reaction was left stirring for 30 min in the dark. The stirring was then turned off, and the AgAu flakes were allowed to sediment for 30 min. The supernatant was removed. If the AgAu flakes were not intended for direct ink formulation (i.e., to be analyzed in SEM or XPS instead), the newly coated flakes were rinsed with water and left to sediment three times before being transferred to 15 mL plastic jar and dried on a hotplate at ca 90 °C. When the newly coated AgAu flakes were intended for ink formulation, they were rinsed three times with DI water and subsequently treated with a mixture consisting of 3 mL of the commercial Baymedix CD102 water-borne PU dispersion (40 wt %) and 37 mL of DI water for 24 h, then rinsed and sedimented twice again with DI water before transfer and drying in the manner described above. Since the empty jar was weighed, the exact weight of the obtained AgAu flakes could be determined by weighing the jar with the flakes again after drying.

### Coated Flake Characterization

Scanning electron microscopy was performed with a Sigma 500 Gemini (Zeiss) at 4 kV using an InLens detector. Energy-dispersive X-ray spectroscopy analysis was performed using a Bruker Quantax EDS. X-ray photoelectron spectroscopy (XPS) measurements were carried out on a custom-build spectrometer (Moses) equipped with an X-ray source from Al Ka radiation (1486.6 eV) and a hemispherical electron analyzer in the analysis chambers, under ultra-high vacuum with the base pressure being lower than 1 × 10^–9^ mbar. To minimize any beam damage on the sample, a large probe area of 1 × 10 mm^2^ and a low X-ray powder of 30 W were employed. All measurement was done at room temperature with a 45° emission angle. The binding energy was calibrated based on the position of the Au 4f_7/2_ peak (84.0 eV) and the Fermi level (0 eV).

### Conductive Ink Formulation

To 959 mg of commercial water-borne Baymedix CD102 PU dispersion (40 wt %), the following was added and carefully mixed by hand, in order: 380 mg of dipropylene glycol, 874 mg of propylene glycol, and 351 mg of 2-hydroxyethyl cellulose solution (10 wt % in propylene glycol). The resulting mixture was added to 6 g of dried AgAu flakes in a 15 mL jar with a lid. The obtained viscous paste was mixed in a SpeedMixer (German Engineering by Hauschild, DAC 600.1 - CM 50) for 8.5 min (30 s at 800 rpm, 8 min at 1800 rpm). The obtained ink was then free from visible particles. For analogue Ag ink, pristine Ag flakes from the bottle were used in the same quantities described for the formulation of the AgAu flakes ink, with the exception of the added propylene glycol amount, which was decreased to 166 mg for the same weight of Ag to achieve a similar viscosity as the AgAu ink.

### Insulating Ink Formulation

To 13.71 g of commercial water-borne Baymedix CD102 PU dispersion (40 wt %) were added and carefully mixed by hand, in order: 4.88 g of dipropylene glycol and 9.39 g of 2-hydroxyethyl cellulose solution (5 wt % in propylene glycol). To the resulting mixture, 19.98 g of Kronos TiO_2_ 2190 was added. The obtained viscous paste was mixed in a SpeedMixer (German Engineering by Hauschild, DAC 600.1 - CM 50) for 8.5 min (30 s at 800 rpm, 8 min at 1800 rpm). The obtained white dielectric ink was then free from visible particles.

### Device Fabrication

Prior to printing, commercial 80 μm A4 laminating pouches from Exibel were laminated onto the PE side of the 50 μm Covestro Platilon U073 TPU substrates with a GMP Lamiart 470LSI office laminator. The laminated substrates were preheated at 120 °C for 30 min in a Termaks type TS 8056 oven to avoid shrinkage during the device process steps. The conductive lines of 1 × 20 mm^2^ (plus 2 × 5 mm^2^ contact pads at the ends) made for the electromechanical testing were screen printed by hand using polyester mesh screens with 77 threads cm^–1^ and 48 μm thread diameter and dried at 110 °C for 30 min to remove solvents. The AgAu conductors for the multilayer tests, the LED demo, and the NFC antenna were screen printed in a flat-bed DEK Horizon 03iX printer using rubber squeegees and metal fillers. The LED conductors were printed in 1 layer and dried in the same manner as described for the conductive test lines. The multilayer test prints and the antenna coils were printed using a 77–48 polyester mesh screen of the same kind as the lines described earlier but dried in a Natgraph Air Force UV Combination Dryer using 3 passes per layer at 110 °C and 1 m min^–1^ (ca 4 m belt length); the number of passes was decided by measuring the resistance in the coil structure after each pass until it had been reduced by less than 5% per pass. For the antenna coils, 3 layers of AgAu ink were printed. A dielectric bridge was printed in 6 layers using a polyester mesh screen with 120 threads cm^–1^ and 34 μm thread diameter and dried using 2 passes per layer in a Natgraph oven at the same temperature and speed as the coil prints. Finally, the microchip footprint and the antenna were printed in 2 layers using a 140–31 mesh screen and dried using 3 passes per layer through the Natgraph oven.

To mount the rigid components (LEDs and microchip, respectively), *Z*-axis conductive tape 9703 from 3M was placed on top of the printed contact pads, and the NHS3100 chip was attached manually using mild pressure. The electrical contacts were further reinforced by the manual addition of a commercial stretchable Ag paste CI-1036, which was dried for 30 min at 110 °C. A mixture of 2-HEC (5 wt % in propylene glycol) and Baymedix CD102 in a 7:43 ratio was used as a globe top to encapsulate only the rigid components and then dried at 70 °C for 5 h. Prior to performing any mechanical testing of the printed NFC antenna, a flexible but inelastic Kapton tape was attached on the bottom side of the substrate in correspondence with the mounted chip.

### Ink and Print Characterization

Rheometric measurements were performed in an Anton Paar Modular Compact Rheometer 102, using a cone-plate system with a diameter of 50 mm and an angle of 1°. The temperature was set to 24 °C ±1. Prior to the flow curve measurements, the samples were presheared at 1 s^–1^ for 30 s. During the flow curve measurements, the shear rate was scaled logarithmically between 10^–2^ and 10^4^ s^–1^. Data was analyzed with RheoCompass v1.24.584-Release software.

The thicknesses of substrates and prints were measured with a Heidenhain ND 287 evaluation unit. Microscope images were obtained with a Leica DM LM microscope and accompanied by LAZ EZ software. Stretching of the samples was performed using an in-house developed stretch tester, consisting of a motorized X-LSQ300A-E01 linear stage (Zaber) with gold-coated contacts for 4-point resistance measurements; the contact pads of the printed test lines were designed to match the setup’s dimensions. Resistances during electromechanical characterization were measured with a Keithley 2701 Ethernet Multimeter data acquisition system. Strain cycling was performed at a speed of 1 mm s^–1^ with a 0.5 s waiting time at the maximum and minimum strain points.

### H_2_O_2_ Corrosion Testing

AgAu and Ag hand-printed lines, 4 of each at a time, were submerged in 15 mL of a 10% solution of H_2_O_2_ for 3 h, and the contact pads were kept above the liquid. Afterward, the prints were carefully dried with pressurized air before they were analyzed.

### Sulfur Contamination Testing

Ag and AgAu hand-printed conductive traces on TPU were placed in a 7 × 10 cm^2^ sealed plastic box. Connections to the exposure chamber were established by drilling holes into the plastic box and connecting Swagelok fittings that were sealed with Blu Tack and poly(tetrafluoroethylene) tape. Gas exposure was controlled by a gas mixer system consisting of mass flow controllers (MFCs) from Bronkhorst High-Tech B.V., AK Ruurlo, Netherlands, with maximum flow rates of 50 (O_2_, SO_2_) or 100 mL min^–1^ (N_2_) connected to PC-controlled sequencing software (home-built Python program). The total flow through the chamber was kept constant at 100 mL min^–1^, and the gas mixture was kept at room temperature. A background mixture of N_2_ and O_2_ with a ratio of 80:20 mL min^–1^ and a constant flow rate of 100 mL min^–1^ was used. Humidity was produced by splitting the dry N2 carrier gas (0% RH: <5 ppm H_2_O for gas from gas cylinders with purity 6.0) into two streams, one of which goes through a water bottle, the so-called “bubbler”, at the exit of which the gas stream can be assumed to have 100% relative humidity. After this, both nitrogen streams (dry and humid) were unified; hence, the humidity could be adjusted by varying the split ratio (relative flow through the dry and wet N_2_ MFCs). The dry nitrogen concentration was then lowered when introducing test gas (20 mL min^–1^ SO_2_ from a 500 ppm SO_2_ in N2 with purity N6.0) to the gas flow to achieve a total gas exposure condition of 100 ppm SO_2_ in a background of simulated air with 40% relative humidity. The AgAu- and Ag-printed samples were exposed for 4 h.

### NFC Antenna Testing

The antenna’s frequency range was assessed using a HP E4407B ESA-E Series Spectrum Analyzer. The design of the antenna was simulated using online software (http://www.circuits.dk/calculator_planar_coil_inductor.htm) and chosen to yield an operation frequency of 13.8 MHz—slightly above the required 13.56 MHz to safeguard against reduced frequency during device stretching.^47^ The practical NFC function was assessed by reading the chip with a mobile phone using the “NFC TagInfo by NXP” app by NXP Semiconductors—the producers of the chip used. The reading range was estimated by measuring the maximum distance between a mobile phone and the antenna during successful reading. To test function during deformation, the antenna was cut out with a free space of 1 cm around each side of the printed patterns. The device was uniaxially stretched by attaching tape to two ends of the TPU substrate. Before, during, and after straining, NFC reading was performed with the mobile phone app, and the frequency response was measured. Furthermore, the antenna was mounted against bare skin, using Tegaderm transparent film roll, and shown to function in a realistic situation where the wrist bends.

## Results and Discussion

### Synthesis of AgAu Flakes

**Figure 1:**
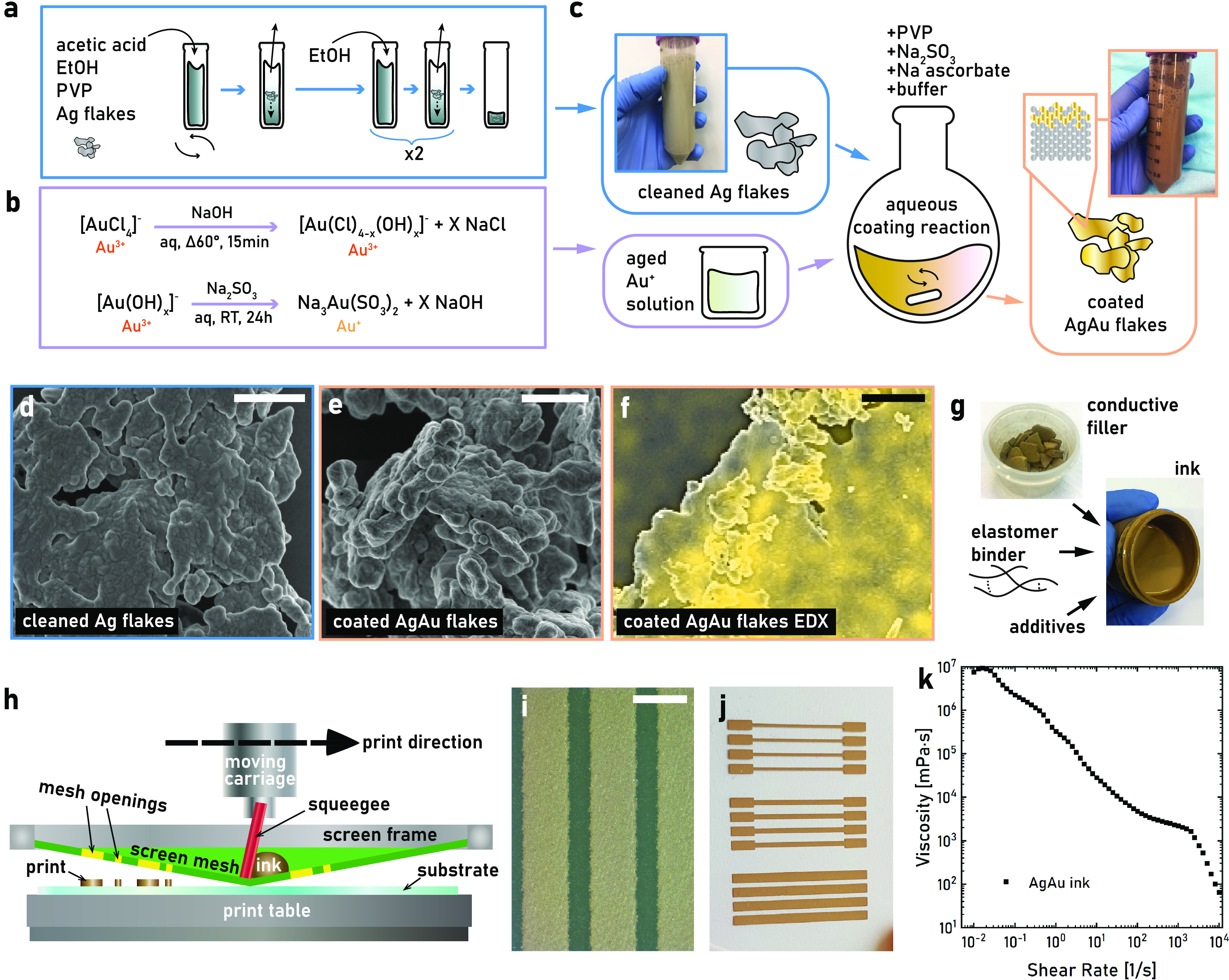
Development of stretchable AgAu ink. (a) The Ag flake cleaning process to enable water dispersion and coating. (b) The Au hydroxylation process followed by reduction and subsequent complex formation with sodium sulfite (Au “aging” process). (c) Overview of the Au coating of Ag flakes process. (d) SEM image showing cleaned Ag flakes. The scale bar is 1 μm. (e) SEM image showing Au-coated Ag flakes. The scale bar is 1 μm. (f) An EDX image showing the presence of Au on AgAu flakes. The scale bar is 1 μm. (g) Stretchable ink formulation including AgAu flakes as a conductive filler, water-based PU as an elastomeric binder, and 2-HEC and co-solvents as additives. (h) Schematic of a flat-bed screen printing process. (i) Microscope image of printed AgAu tracks. The scale bar is 500 μm. (j) Photograph of the printed AgAu ink on the TPU substrate. (k) Flow curve rheology showing that the AgAu ink is shear-thinning.

Commercially available Ag flakes are provided with hydrophobic lubricants adsorbed on the surface to prevent agglomeration.^48,49^ Acetic acid (HAc) was used for removing the lubricant in combination with dispersion agent poly(vinylpyrrolidone) (PVP).^50^ Highly concentrated aqueous HAc solutions produced cleaned flakes but severely corroded the Ag flakes (Figure S1). Ethanol (EtOH) enabled more effective wetting and dispersion of the Ag flakes even at HAc concentrations down to 1%.^51^ This limited the corrosion so that 1.5 g clean high-quality flakes could be produced from a 40 mL EtOH solution (Figure 1a). Subsequently, the flakes were redispersed twice in 40 mL of 99% EtOH to wash away the HAc.

Galvanic displacement coating reactions etch the Ag and tend to produce porous Au coatings, which is undesirable in this context.^52^ The challenge here was therefore to develop a highly concentrated coating process to produce large quantities of material while suppressing the displacement reaction. A concentrated Au coating process of Ag flakes using ascorbic acid (AA) and HAuCl_4_ (252 mM, pH 3) resulted in a significant amount of galvanic displacement reactions in combination with rapid Au nanoparticle formation. Nanoparticle formation can be suppressed by the slow addition of the HAuCl_4_,^25^ but this is undesirable in terms of up-scaling of the coating reaction. Instead, the reactivity of the gold salt was reduced by converting it from HAuCl_4_ (Au^3+^) to a Na^3^Au(SO_3_)^2^ (Au^1+^) sulfite complex (Figure 1b),^44,53^ which lowers the reduction potential from 0.85 V vs SHE to 0.11 V.^54^ The addition of sulfite to an acidic HAuCl_4_ precursor at room temperature led to rapid nanoparticle formation (Figure S2). To avoid this, the HAuCl_4_ precursor was converted into {Au(Cl)_4–*x*_(OH)_x_}^−^ 0 ≤ *x* ≤ 4 to lower the reduction potential of the complex (Figure 1b).^43,52,55,56^ Enough NaOH was added to a 252 mM HAuCl_4_ solution to reach pH ∼9, after which the solution was heated to 60 °C for 15 min.^57^ Next, hydroxylated Au and Na_2_SO_3_ were mixed in a molar ratio of 1:3, and reaction times from 2 min to 5 days were evaluated by Ag flake coating trials, which showed that 24 h of aging in the dark (Figure S4) gave the best surface morphology with low roughness (Figure S3). The pH was kept >9 by the addition of an alkaline NaOH–glycine buffer to favor the reduction process over displacement (Figures S5, S6).^53,58^ The final reaction recipe reliably produced Au coatings of good coverage, which was not the case for the non-buffered reactions where the results were very sensitive to variation in the reaction conditions, such as the speed of addition of the Au growth solution (Figures 1c, S7). Glycine can also act as an anionic capping agent for Au nanoparticles at high pH and may thus affect the morphology of the coatings.^59^

Scanning electron microscopy (SEM) imaging was used to assess the Au coverage of the flakes (Figure 1d,e). Poor coating conditions could be clearly identified in SEM via openings in the brighter Au layer or with Au islands merely dotting the Ag bulk surface (Figure S8). The optimized process yielded uniform coatings without cracks and with different surface textures compared to the cleaned or pristine Ag flakes (Figure S9). Energy-dispersive X-ray spectroscopy (EDX) confirmed the presence of Au on the flakes (Figure 1f), with line scans giving a normalized mass ratio of 14% Au and 86% Ag (Figure S10). This is close to the mass ratio within the reaction mixture (12% Au, 88% Ag), although EDX has limited quantitative accuracy. The composition of the top portion (≈5 nm) of the flakes was further studied by X-ray photoelectron spectroscopy (XPS), which showed a mass ratio of 41% Au and 59% Ag (Figure S11). This indicates that the surface is composed of an AuAg alloy, likely due to some displacement reactions during the coating. The optimized process coated 1.5 g of Ag flakes per 200 mL solution, which is more than a 5000% increase in Ag concentration in comparison to previous work on nanowires.^53^ This is of utmost importance for the intended application, as flake quantities of >5 g are typically needed even for test prints. The high concentration and excellent process stability should enable further up-scaling of the coating process, thereby enabling the production of hundreds of grams of flakes, which is desirable for screen printing inks.

### Ink Formulation and Printing

The developed ink was based on AgAu flakes and a soft medical-grade PU water dispersion (Baymedix CD102 by Covestro), which formed a solid film upon drying (Figure 1g). The printability of the inks was tested by screen printing them by hand in simple lines using nylon mesh screens with 77 threads cm^–1^ and 48 μm thread diameter (Figure 1h). The use of such rough mesh resulted in prints with coarse edges but was deemed preferable to produce thick enough prints to achieve acceptable sheet resistance (Figures 1i, S12). To slow down the drying of the water-based ink, the addition of three harmless high-boiling-point solvents was evaluated: propylene glycol (PGly, bp 188 °C), dipropylene glycol (DGly, bp 230 °C), and tripropylene glycol (TGly, bp 273 °C). Both PGly and DGly evaporated during drying for 30 min at 110 °C, while TGly remained even after 60 min drying. Therefore, PGly and DGly were chosen as high-boiling-point solvents for the ink. The viscosity of the ink was controlled by the addition of the rheological modifier 2-hydroxyethyl cellulose (2-HEC) dissolved in PGly. High amounts of 2-HEC (≥14 vol % in the dry print) degraded the elastic properties of the printed film, causing visible cracks when elongated, while amounts ≤6 vol % (in the dry film) did not have the desired effect on ink rheology and print quality. Hence, 6 vol % (in the dry film) of 2-HEC was chosen for the inks.

Another challenge when formulating the ink was the AgAu flake dispersion in the prints, which is crucial for the performance of stretchable composite conductors.^26^ The dispersibility of the AgAu flakes was improved by prior treatment with water-dispersed PU, sedimentation, and DI water washing of the flakes. The procedure improved flake dispersion in the printed structures and lower sheet resistance, supported by optical microscopy and electromechanical cycling tests, respectively (Figures S12, S13). Finally, the AgAu filler–elastomer ratio was optimized with respect to electromechanical properties. A high filler ratio tends to give high initial conductivity but poor mechanical properties with quickly deteriorating performance under strain. AgAu loadings between 25 vol % and 65 vol % were evaluated to optimize the performance of the AgAu-PU ink, but 45 vol % was the lowest limit for good printability and conductive films in this mixture. The optimal loading was 60 vol % AgAu flakes (dry film) (Figures 1j, S14, Table S1). The final ink formulation consisted of a weight of 70.1% AgAu flakes, 11.2% PU dispersion (40 wt % in water), 10.2% propylene glycol, 4.4% dipropylene glycol, and 4.1% 2-HEC solution (10 wt % in propylene glycol). The rheology of the ink was assessed via a flow curve measurement, which showed that the ink was shear-thinning, a requirement for well-performing screen printing inks (Figure 1k).

**Figure 2:**
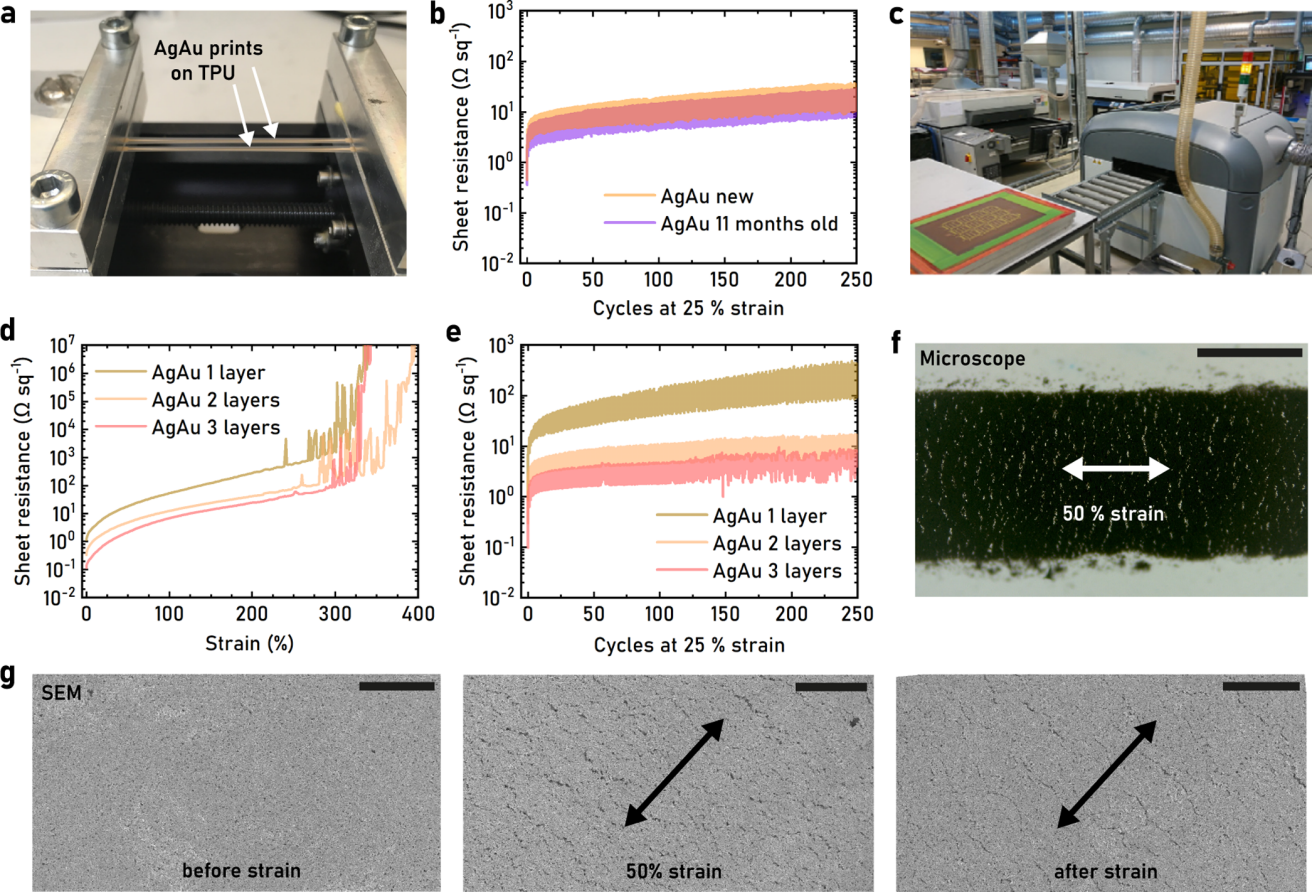
Characterization of stretchable printed conductors. (a) AgAu prints mounted and strained in the 4-probe measurement linear strain setup. (b) Change in sheet resistance during 25% strain cycling for new and 11-month-old AgAu prints. (c) Photograph of the screen printing facility at Printed Electronics Arena, Norrköping, including a DEK Horizon 03iX and a Natgraph belt oven. (d) Resistance vs strain measurement until failure for AgAu printed conductors. (e) Change in sheet resistance of AgAu prints during 25% strain cycling for 1, 2, and 3 layers. (f) Backlight microscope imaging of a AgAu print during 50% strain. Scale bars are 500 μm. (g) SEM images of AgAu prints before, during 50% strain, and after strain, respectively. Scale bars are 100 μm.

The selection of substrate and compatibility between the ink and the substrate are keys to the successful development of printed electronics. A biocompatible stretchable TPU film (Covestro, Platilon U073) of 50 μm in thickness was chosen as the printing substrate. The TPU film was provided with a non-elastic polyethylene (PE) carrier foil; however, the carrier became mechanically unstable at desirable drying temperatures (up to 120 °C) and was not rigid enough to prevent deformation during the printing process. Therefore, a commercial 80 μm inelastic plastic foil (Exibel, A4 laminating pouches) was laminated onto the PE foil prior to printing, and a preheating step (120 °C for 30 min) was added to minimize deformations during the ink drying steps. The TPU film had a specified melting point of 160–200 °C, but it started to soften significantly already at 140 °C, further motivating the use of lower curing temperatures. At the end of the fabrication process, the laminated foil was peeled off together with the PE layer. Hand-printed conductors consisting of 1 × 20 mm^2^ lines, with additional 2 × 5 mm contact pads on each side, were used for initial stretching tests (Figure 1j). The samples were clamped in a four-probe setup for resistance measurements during uniaxial stretching of the samples (Figure 2a). AgAu prints of 12.6 ± 1.0 μm thickness (*n* = 5) reached an initial sheet resistance of 0.27 ± 0.03 Ω sq^–1^ (*n* = 8, ≈3000 S cm^–1^), which increased to 9.00 ± 1.89 Ω sq^–1^ (*n* = 4) at 50% strain (Figure S15). Corresponding Ag prints made from analogue ink with untreated Ag flakes had a similar strain behavior but a lower initial sheet resistance of 0.07 ± 0.02 Ω sq^–1^ (*n* = 7). Strain cycling to 10, 25, and 50% strain at 1 mm s^–1^ was performed for the AgAu prints and the Ag prints (Figure S15). The AgAu prints outperformed the Ag prints in terms of relative resistance change (*R*/*R*_0_), indicating that the gold coating improved the filler–elastomer interactions within the film. After 250 cycles, the AgAu sheet resistance for the hand-printed samples at 10, 25, and 50% strain was 3.6, 12.5, and 51.4 Ω sq^–1^, respectively. The ambient long-term stability of AgAu prints was tested by comparing prints from the same batch when they were newly printed with those stored at 50% RH and 20 °C for 11 months; no degradation in electromechanical behavior was found (Figures 2b, S15). In an effort to improve the film properties, the AgAu ink was also printed in a semi-automatic DEK Horizon 03iX screen printer (Figure 2c), and comparisons were made between 1-, 2-, and 3-layer prints (Figure 2d,e). The 3-layer prints measured 35.7 ± 1.7 μm in thickness (*n* = 9) and reached an initial sheet resistance of only 0.033 ± 0.002 Ω sq^–1^ (*n* = 7), which corresponds to ≈ 8500 ± 400 S cm^–1^, indicating that multilayer deposition improved the film properties beyond the decrease in sheet resistance due to increased thickness, a phenomenon that has previously been observed.^60^ SEM imaging of focused ion beam cuts in AgAu prints reveals that the films have a rather porous internal structure (Figure S16). The additional solvents and mechanical forces on the films in multilayer printing processes can therefore affect the film morphology, which could explain the observed increase in conductivity. The initially printed films showed a smooth surface with the filler particles clearly visible (Figure 1i). Upon stretching, cracks became visible in the film when viewed through a microscope and SEM (Figure 2f,g), correlating to the increase in resistance observed during strain. Once the strain was released, many of the cracks closed and the resistance recovered, although to a substantially higher value than the initial resistance.

**Figure 3:**
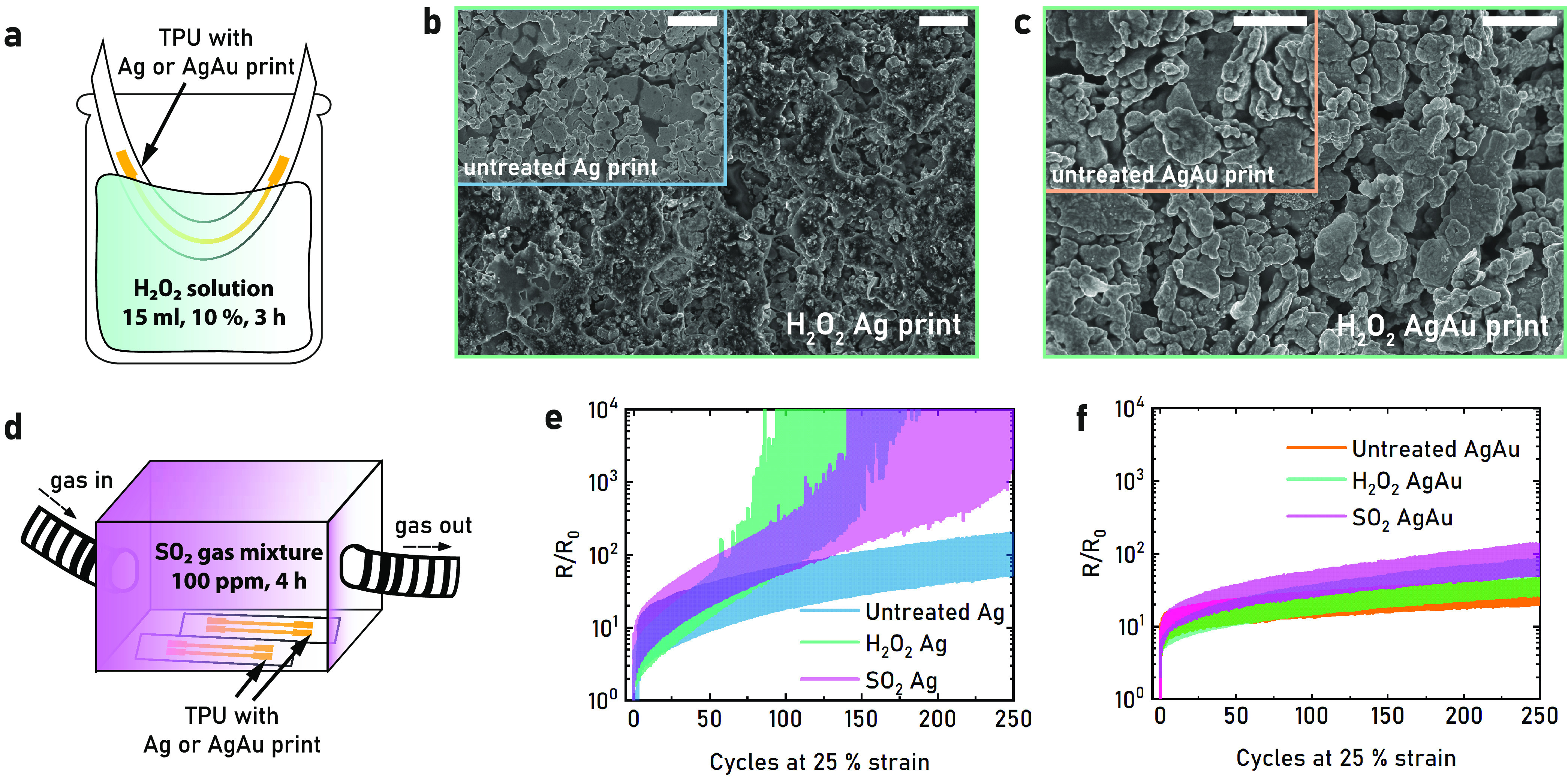
Corrosion stability of printed stretchable conductors. (a) Schematic of a print submerged in 10 vol % H_2_O_2_ with the contact pads above the surface. (b) SEM image of Ag print treated with H_2_O_2_, with untreated Ag print as an inset. Scale bars are 2 μm. (c) SEM image of AgAu print treated with H_2_O_2_, with untreated AgAu print as an inset. Scale bars are 2 μm. (d) Schematic of prints exposed to the SO_2_ gas mixture. (e) Relative resistance for cycling at 25% strain of untreated Ag prints compared to Ag prints treated with H_2_O_2_ and SO_2_. (f) Relative resistance for cycling at 25% strain of untreated AgAu prints compared to AgAu prints treated with H_2_O_2_ and SO_2_.

Corrosion, oxidation, and tarnishing are some of the potential issues of using non-noble metals as conductive fillers in real-life applications. Ag, despite sometimes being denoted as a noble metal, is susceptible to these issues as well. The corrosion resistance of hand-printed single-layer AgAu and Ag lines was evaluated after submerging the prints in 10 vol % H_2_O_2_ for 3 h (Figure 3a). SEM images of H_2_O_2_-exposed Ag prints revealed severe degradation of the film, with flake shapes barely recognizable (Figure 3b). In contrast, SEM images showed little change in the exposed AgAu prints, with only a low density of small particles present on the AgAu flake surfaces (Figure 3c). The initial sheet resistance of Ag prints went up from 0.07 ± 0.02 to 1.06 ± 0.13 Ω sq^–1^ (*n* = 2) after H_2_O_2_ exposure, while the AgAu resistance changed from 0.27 ± 0.03 to 0.93 ± 0.15 Ω sq^–1^ (*n* = 2). The exposed Ag prints showed severely degraded performance during strain cycling (Figure 3e, S17), while the cyclic performance of the AgAu prints was better preserved (Figures 3f, S17). To simulate tarnishing by sulfuric compounds, the samples were exposed to 100 ppm SO_2_ for 4 h in a sealed box (Figure 3d). SEM imaging of the SO_2_-exposed flakes revealed no evident alteration in the surface appearance of the flakes (Figure S18). Despite the lack of visible degradation by the SO_2_ gas, the initial sheet resistance of the Ag prints increased from 0.07 to 0.17 ± 0.02 Ω sq^–1^ (*n* = 2), while the AgAu prints increased from 0.27 to 0.44 ± 0.06 Ω sq^–1^ (*n* = 2). Also, here, the cyclic performance of the Ag prints was severely degraded (Figure 3e), while the AgAu prints were better preserved (Figure 3f). Overall, the poor cyclic performance of exposed Ag is consistent with increasing contact resistance between flakes due to insulating layers, while the Au coating prevents the formation of insulating layers to a large extent. Some decreased performance from the AgAu prints was expected, as the XPS characterization showed that the coating is in the form of an AgAu alloy, also with the possibility of nanoscopic pores and local Ag_2_S formation.^61^ The presence of Ag on the surface of the coated flakes was also detected in cyclic voltammograms of printed Ag and AgAu films, where clear oxidation and reduction peaks for Ag and Ag and Au, were visible (Figure S19). Altogether, the results still suggest that the level of Au coating on Ag flakes achieved by our method effectively protects the printed structures from corrosion and tarnishing, thereby preserving the performance over time.

### Stretchable Corrosion-Resistant Wearables

**Figure 4:**
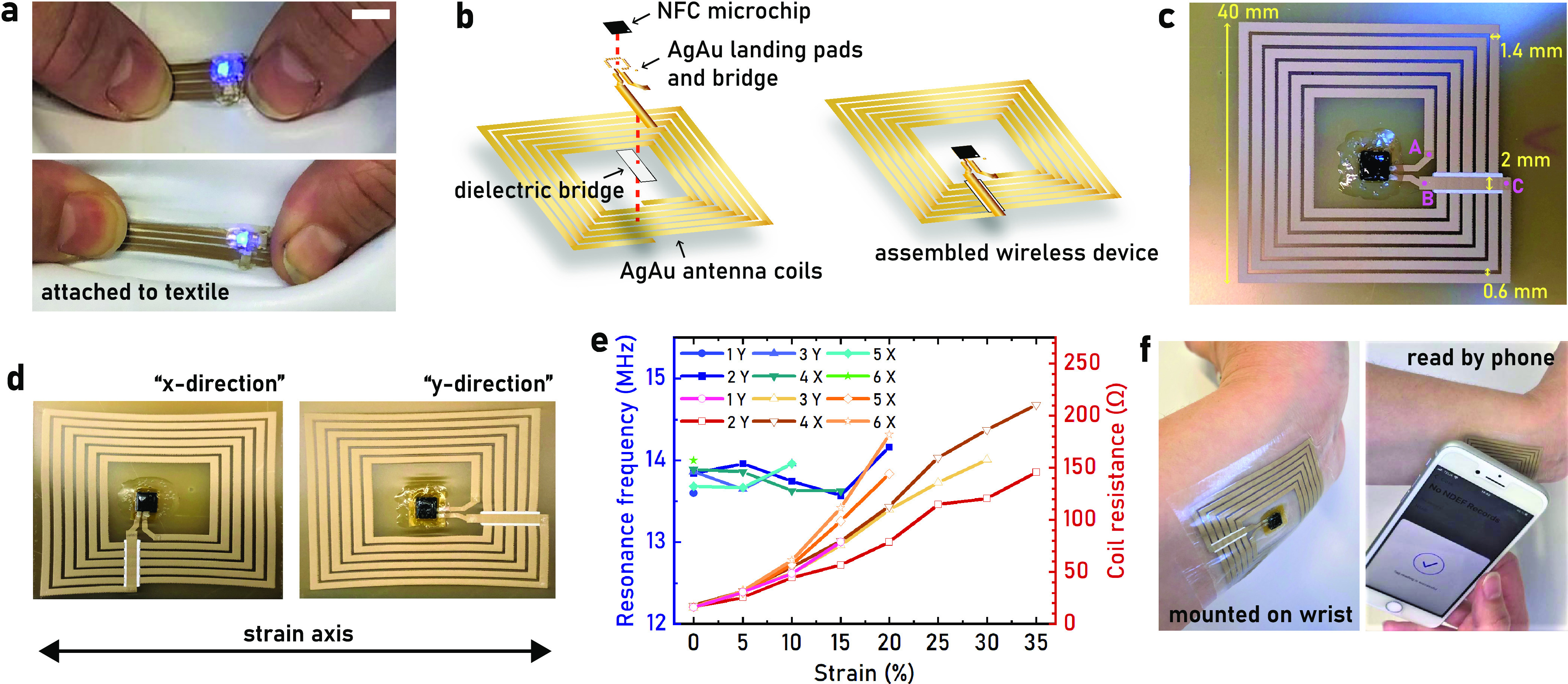
Stretchable corrosion-resistant wearables. (a) A LED is mounted onto the AgAu prints and the circuit, including the TPU substrate, is hot pressed onto a stretchable textile. The LED is functional during repeated stretching to >100% strain. The scale bar is 1 cm. (b) Schematics of the stretchable NFC device stack. (c) Photograph of a printed hybrid NFC circuit base on the AgAu ink, with probing points A–C for resistance measurements marked. (d) The AgAu NFC device stretched to 30% perpendicular to the bridge (*x*-direction) and to 35% along the bridge (*y*-direction). (e) Coil resistance of individual antennas increasing with increasing uniaxial strain in either the “*x*-direction” or the “*y*-direction”, as explained in (d), together with recorded resonance frequencies. (f) The AgAu NFC device on a biocompatible substrate mounted on the skin and conforming when stretched while still being read by a mobile phone application.

A stretchable LED circuit was developed to demonstrate the applicability of the AgAu ink. Interconnects and mounting pads for LEDs were printed onto TPU substrates using the semi-automatic DEK Horizon 03iX screen printer. The LEDs were mounted onto two interconnects using 3M Z-conductive tape and additional commercial silver ink (CI-1036) to reinforce the connections. The mounting was mechanically reinforced by the addition of a viscous mixture of Baymedix CD102 PU dispersion and 2-HEC (5% w/w in propylene glycol) in a 43:7 weight ratio around the LED. The device was attached onto a stretchable textile using the 3M Tegaderm transparent film and hot-pressing for 20 s at 175 °C, with the heating element contacting the textile side. The LED circuit was robust and could be stretched >100% strain repeatedly while remaining functional (Figures 4a, S20, Video S1).

Wireless communication is a key feature in emerging wearable technology. To explore the capabilities of the developed AgAu ink within this application area, screen-printed hybrid self-powered NFC circuits were developed and characterized (Figure 4b). The targeted resonance frequency of the circuit is 13.56 MHz, which requires that the inductance of the antenna is matched to the input capacitance (50 pF) of the used NFC microchip. As the resonance frequency was expected to shift upon stretching of the antenna,^49,62,63^ a resonance frequency of 13.8 MHz was targeted by the use of a single-layer planar spiral coil inductor calculator online tool.^47^ The resulting antenna design measured 40 × 42 mm^2^ with 6 turns, a line width of 1.4 mm, and a line separation of 0.6 mm (Figure 4c). As the sheet resistance of single-layer prints of the AgAu ink was too high (0.27 Ω sq^–1^) to get a good reading distance, 3 layers of AgAu ink were printed (0.033 Ω sq^–1^). The circuit required a bridge from the outer part of the coil to its interior, which was implemented by first printing an insulating layer followed by printing a AgAu interconnect. The developed stretchable insulating ink formulation was inspired by the AgAu ink, with TiO_2_ powder (Kronos 2190, 47 dry vol %) replacing the AgAu flakes to preserve good rheological properties. To achieve adequate electrical insulation, 6 layers of the ink were printed, yielding a thickness of 30 ± 1 μm (*n* = 3). A finer screen was used to print the bonding pads (250 μm lines, 500 μm pitch) of the used QFN packaged NFC chip (NHS3100, NXP Semiconductors), with two printing layers resulting in 16.8 ± 2.9 μm film thickness (*n* = 4). The NFC chip was bonded in the same way as the previously described LEDs, with 3M Z-conductive tape and commercial silver ink (CI-1036). The mounting was mechanically reinforced with a water-dispersed PU mixture and Kapton tape on the backside of the TPU substrate (Figure S21).

The printed NFC circuit resonated at a frequency of 13.8 ± 0.1 MHz (*n* = 7) and had a quality factor (Q factor) of 2.7 ± 0.4 (*n* = 5), which resulted in a 2 cm reading distance with an iPhone 8 mobile phone (Figure S22). The coil resistance (excluding the bridge) was measured to be 16.45 ± 0.74 Ω (*n* = 7), while the bridge had a resistance of 0.87 ± 0.19 Ω (*n* = 7), which thus was not limiting in the relaxed state. The NFC circuit was uniaxially stretched in two directions to evaluate its performance under strain (Figure 4d). The well-functioning NFC circuits could be read out by the mobile phone at 30% strain, while all initially functional circuits remained operational to at least 15% strain. The variation in performance likely comes from differences in coil resistance under strain, as some printed coils experienced a sharp increase in resistance even at moderate strains (Figure 4e). The resonance frequency did not shift significantly at moderate strains, and it was not possible to accurately determine for strains >15% (Figures 4e, S23). The coil resistance increased by a factor of 4.7 and 3.4 for 15% strain in the X- and *y*-directions, respectively (Figure S24). The bridge experienced a larger difference in directional resistance increase (factor 3.7 and 0.5) but was not limiting the overall performance of the circuit. To demonstrate the applicability of the developed printed NFC circuit, it was attached onto the wrist using a medical-grade adhesive tape from 3M. The NFC patch was stretchable enough to conform well to the skin without inducing any discomfort and remained functional under the maximum movement of the wrist (Video S2, Figure 4f). The printed conductors thus fulfill the basic requirements for wearable patches in terms of performance.

## Conclusions

There is an ever-growing need for conformable electronic devices that are not only easy to wear and use but also stable against physical manipulation and environmental factors. In these pursuits, costs and efficient production become crucial factors for success. Here, we have developed a scalable process for chemically coating Ag flakes with a thin Au layer to improve stability against corrosion and tarnishing. Screen printing inks were developed based on the AgAu flakes, water-dispersed PU and green solvents, and printed stretchable conductors showed an initial conductivity of ≈3000 S cm^–1^. Three-layer prints showed a dramatic increase in conductivity to ≈8500 S cm^–1^, which is comparable to the best stretchable Ag flake conductors in the literature.^20,64,65^ Printed conductors based on the AgAu flakes had good resistance to corrosion (H_2_O_2_ solution) and tarnishing (SO_2_ gas), while pure Ag-based conductors failed under the same conditions. To demonstrate the applicability of corrosion-resistant printed stretchable conductors, stretchable LED and NFC circuits were developed based on the AgAu conductors. The NFC circuit could be attached directly onto the skin and remain functional during stretching induced by wrist movements. To further improve the operational strain range of the printed circuits, the conductor performance under strain should be improved by tuning the flake-filler morphology and interactions. In this work, we employed environmentally friendly solvents for the processing, which limited the type of elastomer systems that could be used. By changing the solvents and elastomers, it is likely possible to improve the performance of the printed conductors so that it becomes comparable to that of non-screen printed stretchable conductors. At the time of writing, 1 kg of Ag flakes costs ≈1500 USD, while the gold for coating 1 kg of Ag flakes costs ≈7700 USD. It is thus important to further optimize the process to reduce gold consumption, ideally by a factor of 5, to reach a comparable cost to the Ag. We believe that the combination of excellent stability, scalable coating, and high-throughput printing makes the demonstrated materials and processing attractive for the commercial production of wearables.

## Data Availability

The data that support the finding of this study are available from the corresponding authors upon reasonable request.

## Supporting Information Available

Photographs and SEM images of prepared materials and devices, and characterization (EDX, XPS, resistance-strain, FIB-SEM, NFC antennas) (PDF)
