# Supplementary material for: Screen-Printed Corrosion-Resistant and Long-Term Stable Stretchable Electronics Based on AgAu Microflake Conductors
Source: ACS Appl Mater Interfaces. 2023 Feb 23;15(9):12372–82. doi: 10.1021/acsami.2c22199 (PMC9999352; doi:10.1021/acsami.2c22199)
Supplement: Supplementary file 1 — am2c22199_si_001.pdf [file am2c22199_si_001.pdf]

Supporting Information

Screen-Printed Corrosion-Resistant and Long-Term Stable Stretchable Electronics Based on AgAu Microflake Conductors

Ulrika Boda[1,3], Jan Strandberg[1], Jens Eriksson[2], Xianjie Liu[3], Valerio Beni[1], Klas Tybrandt[3]**

[1] Bio and Organic Electronics Unit, Department of Smart Hardware, Digital Systems Division, RISE Research Institutes of Sweden AB, 602 21 Norrköping, Sweden

[2] Department of Physics, Chemistry and Biology, Linköping University, 581 83 Linköping, Sweden

[3] Laboratory of Organic Electronics, Department of Science and Technology, Linköping University, 602 21 Norrköping, Sweden

E-mail: valerio.beni@ri.se and klas.tybrandt@liu.se

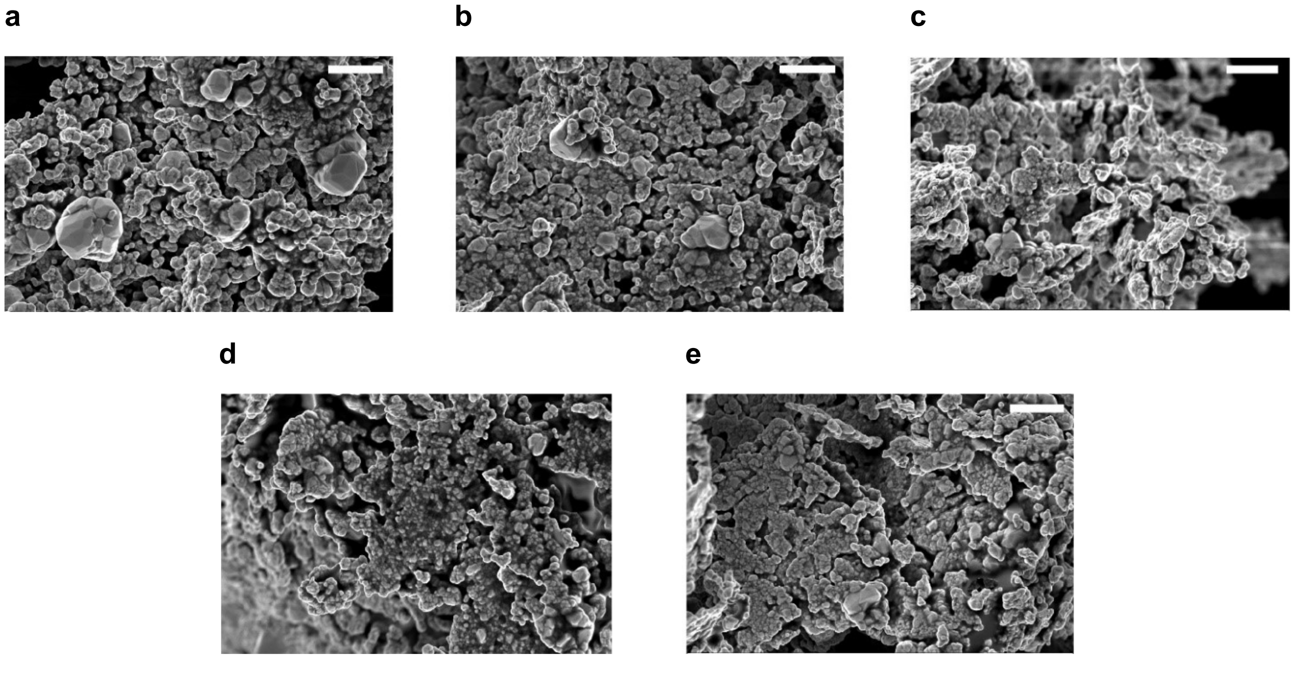

Figure S1. Ag cleaning trials in DI water dispersions using 30 mL of 80% HAc in water and 10 mL of 25 % PVP in water, including time for sedimentation. Scale bars are 2 µm. a) 30 h total cleaning time. b) 18 h total cleaning time. c) 12 h total cleaning time. d) 8 h total cleaning time. e) 1 h total cleaning time.

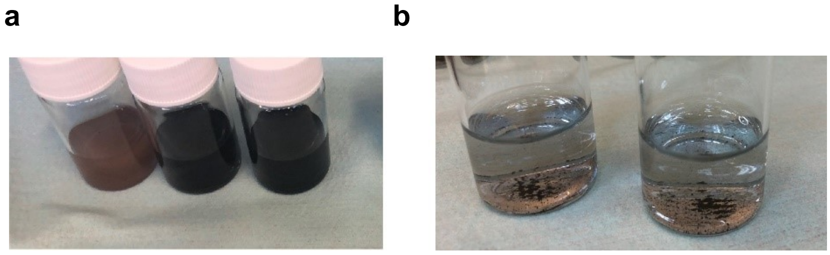

Figure S2. Au aging trials. a) To the bottles was first added $HAuCl_4$ and then Na_2SO_3 and lastly NaOH, after 15 min, 2 min and a few seconds respectively. The opaque contents indicate nanoparticle formation of metallic Au. b) To $HAuCl_4$ was first added NaOH, and then Na_2SO_3 before the first ingredients had finished reacting, yielding large particle formation but not as vivid nanoparticle dispersion in the medium.

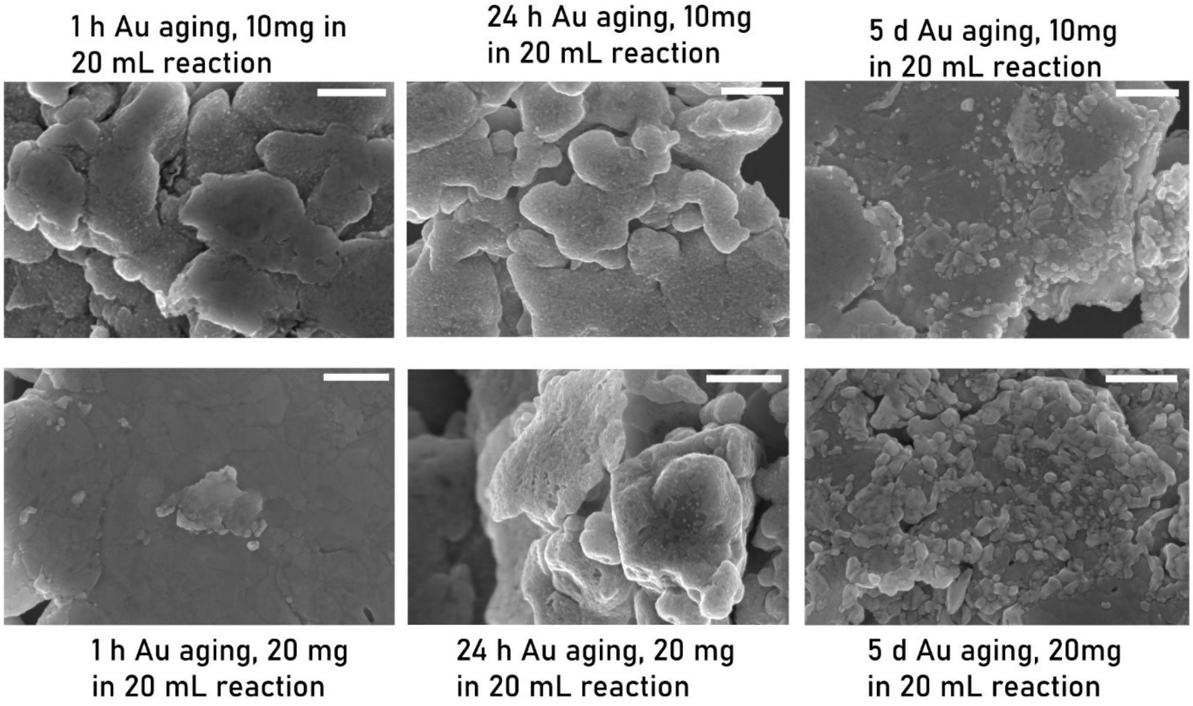

Figure S3. Au on Ag coating reactions run after $HAuCl_4$ solution had aged for 24 h or 120 h, using 5, 10 or 24 mg Ag flakes. Scale bars are 500 nm.

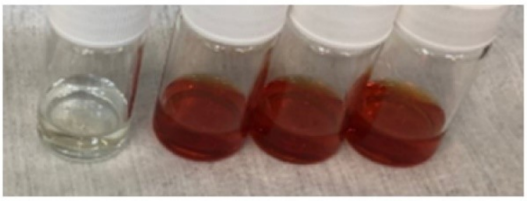

Figure S4. Tests with Au aging solutions show that the process is light sensitive; the three bottles on the right were aged in ambient lighting while the leftmost was aged in darkness.

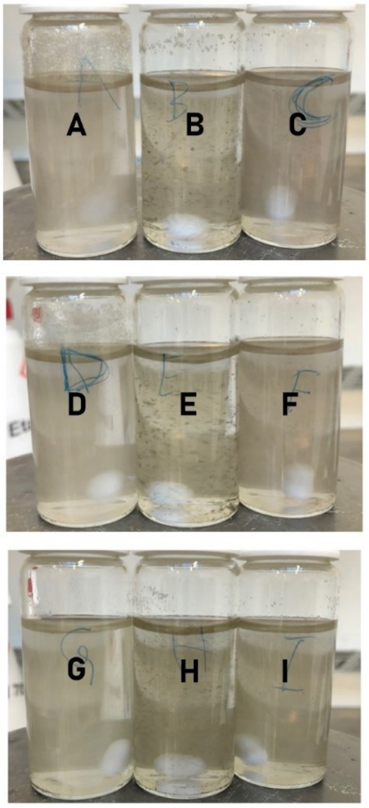

Figure S5. Au on Ag coating reaction trials with (from left) buffer at pH 8.6, 9.6 and 10.6 and (from top to bottom) total PVP concentrations of 0.05, 0.5 and 5 wt%.

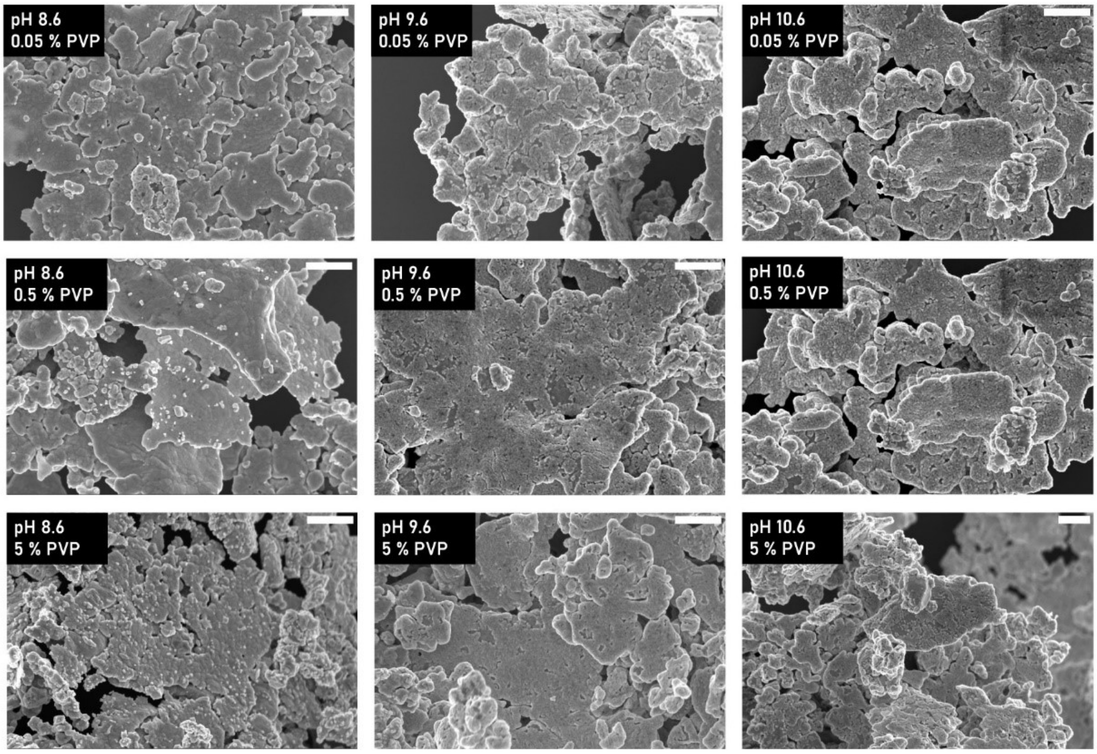

Figure S6. SEM of the pH and PVP tests. Top to bottom: PVP concentrations of 0.05 wt%, 0.5 wt% and 5 wt%. Left to right: pH 8.6, 9.6 and 10.6. Scale bars are 1 μm.

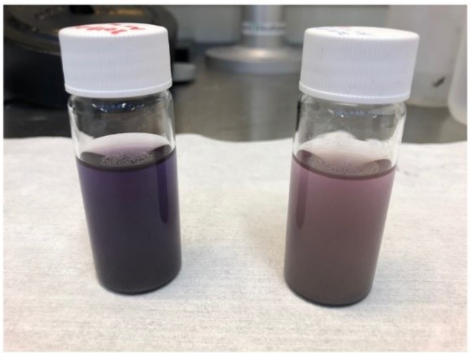

Figure S7. Following the recipe of Choi et. al with flakes instead of NWs; reactions proceeded without addition of buffer. Purple (left bottle): aged Au solution was poured in over less than 1 s. Pink (right bottle): aged Au solution was dripped in over the course of a minute. Reaction fluid volume: 20 mL.

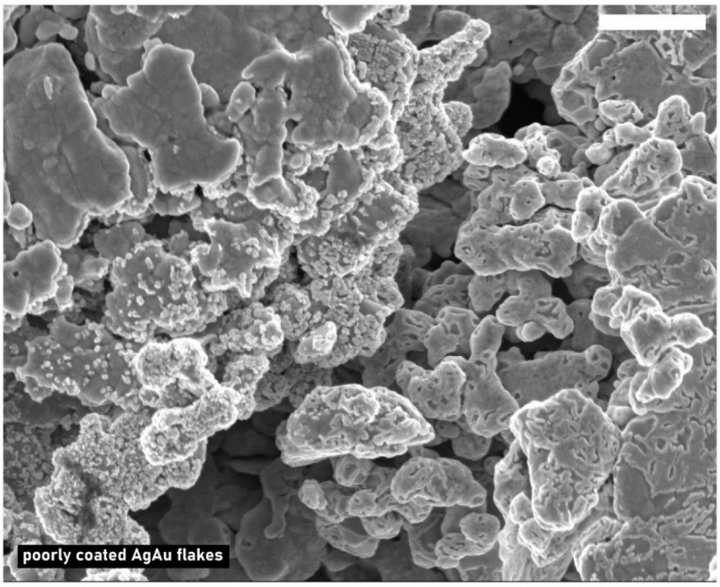

Figure S8. Example of Ag flakes with poor Au coating, as seen in SEM. Scale bar is 1 µm.

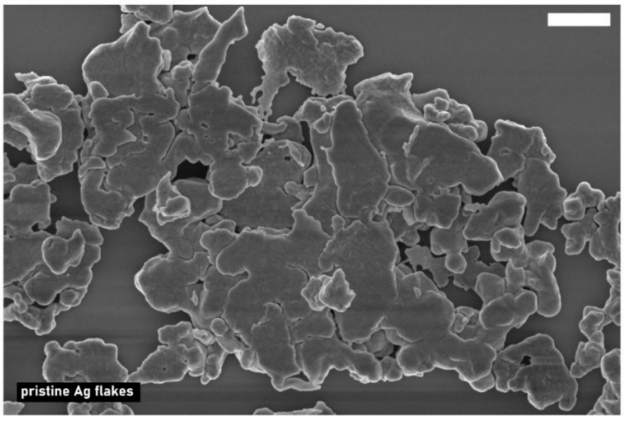

Figure S9. SEM imaging of pristine Ag flakes from the bottle.

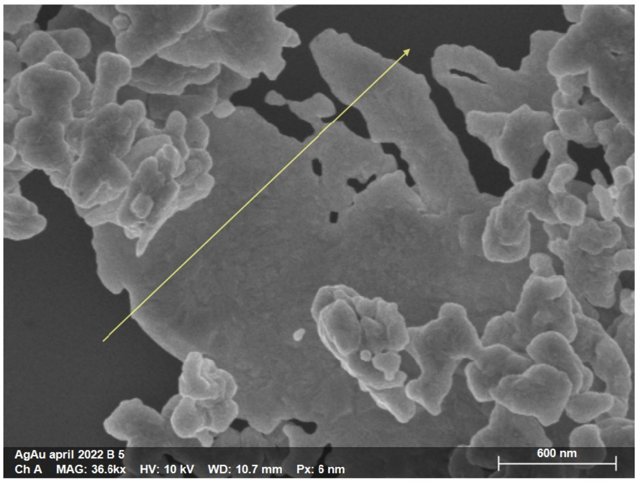

Name	Date	Time	HV [kV]	Mag	WD [mm]
AgAu april 2022 B 5	21-Apr-22	15:23:19	10.0 keV	36553x	10.7 m

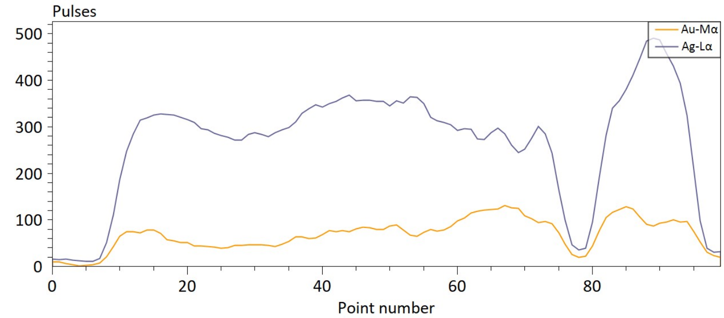

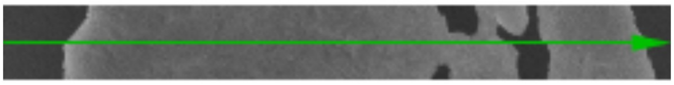

Element	At. No.	Netto	Mass [%]	Mass Norm. [%]	Atom [%]	abs. error [%] (1 sigma)	rel. error [%] (1 sigma)
Gold	79	11879	11.17	13.64	7.96	0.46	4.14
Silver	47	62263	70.74	86.36	92.04	2.40	3.39
		**Sum**	**81.91**	**100.00**	**100.00**		

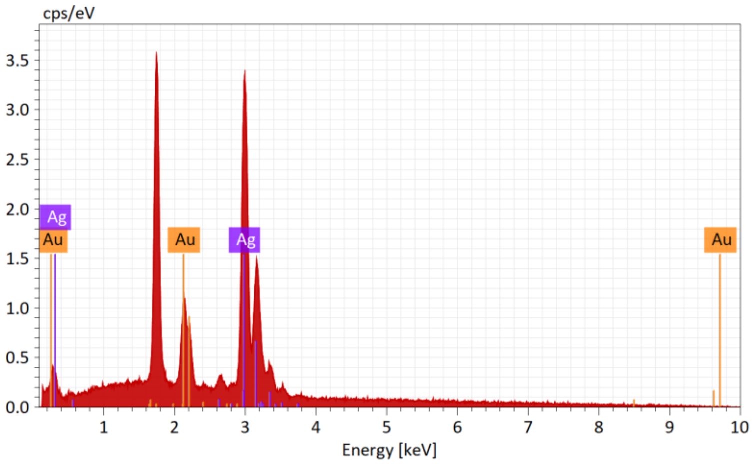

Figure S10. EDX assessment of AgAu flakes. Line scan and quantification spectrum of an Ag flake coated with Au on all sides. The largest peak seen in the spectrum is from the silicon plate the flakes were sampled on.

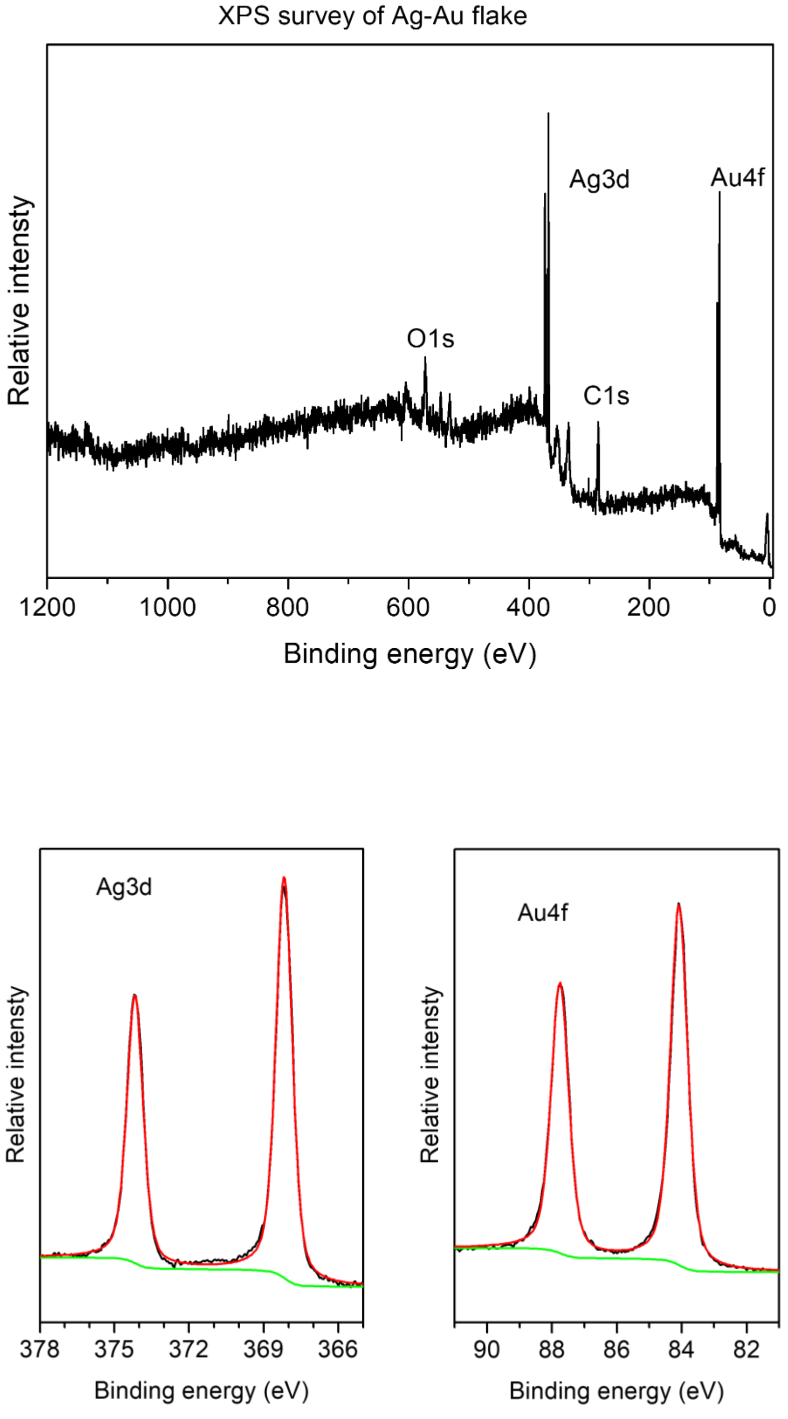

Figure S11. XPS survey on surface of AgAu flakes. The atomic ratio of Ag to Au is 2.625, which indicates that 27.6 % Au and 72.4 % Ag atoms were detected by XPS on the surface, or 41.0 wt% Au and 59.0 wt% Ag.

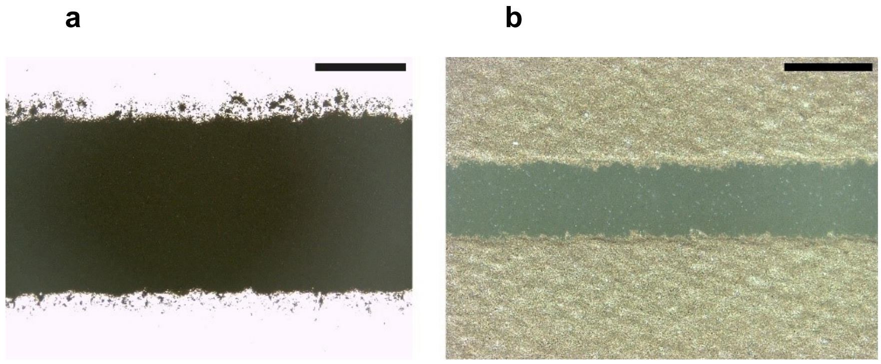

Figure S12. Microscope pictures of AgAu printed line displaying no pinholes. Scale bars are 500 um. a) With backlight. b) With natural light and no backlight.

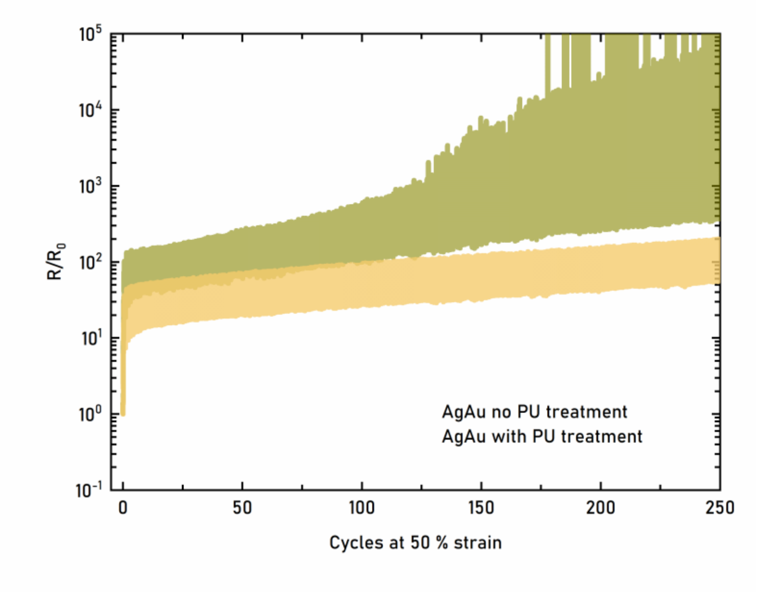

Figure S13. Resistance change measured during strain cycling at 50 % max strain of AgAu ink made from Baymedix-treated AgAu flakes compared to ink made with flakes without the treatment.

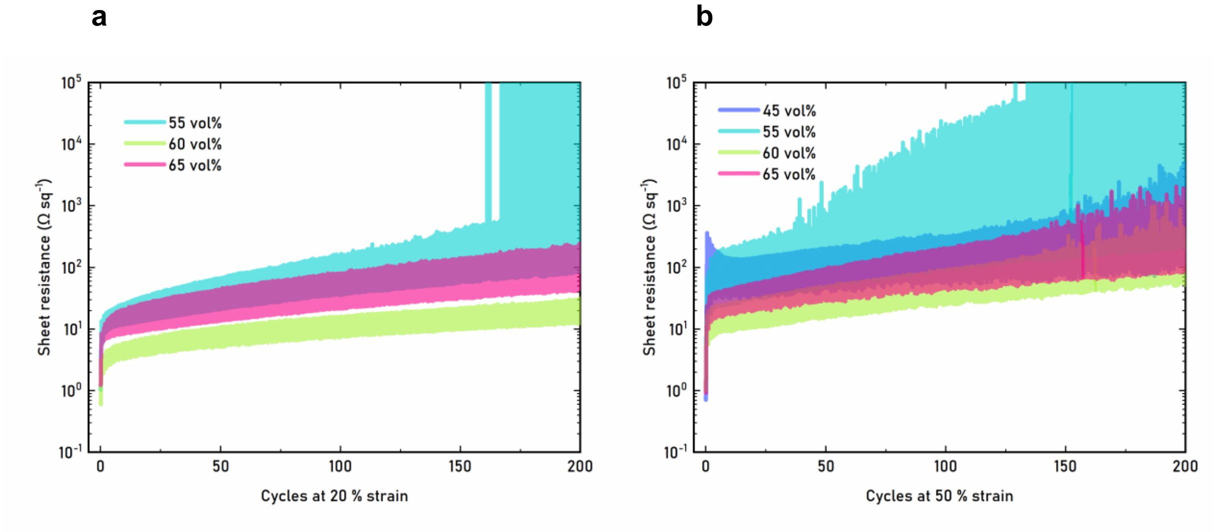

Figure S14. Resistance measured during strain cycling at (a) 50 % and (b) 20 % max strains of AgAu ink made from Baymedix-treated AgAu flakes between 45 and 65 vol% filler loading in the dried print.

AgAu filler loading (vol %)	Average initial sheet resistance (Ω/□)
45	0.65
55	0.97
60	0.27
65	0.98

Table S1. Average initial sheet resistances across test prints of various AgAu filler loadings.

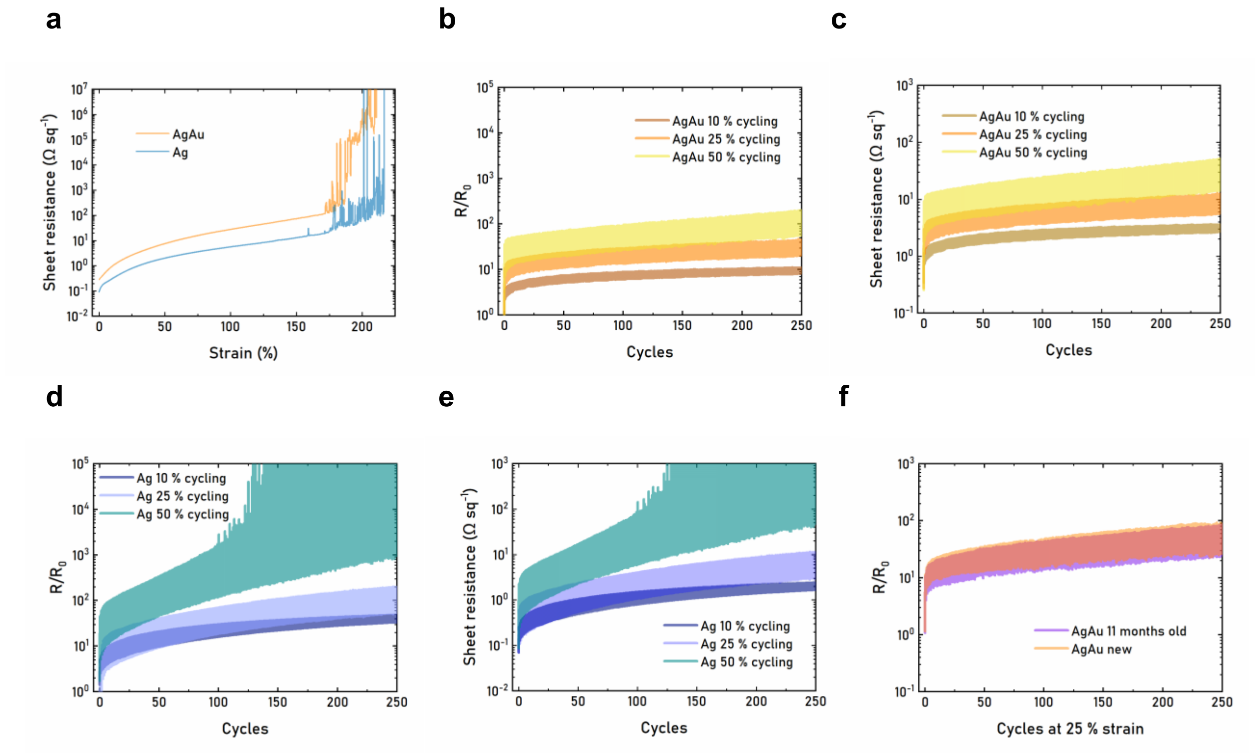

Figure S15. Strain-resistance measurement results of printed AgAu and Ag tracks. a) Resistance vs strain measurement until failure for Ag and AgAu printed conductors. b) Relative change in resistance of AgAu prints at 10 %, 25 % and 50% strain cycling, respectively. c) Printed AgAu sheet resistance variation during 10, 25 and 50 % maximum strains. d) Relative change for Ag prints during 10, 25 and 50 % maximum strains. e) Printed Ag sheet resistance variation during 10, 25 and 50 % maximum strains. f) Relative change for AgAu during 25 % strain cycling for new prints compared to prints aged 11 months.

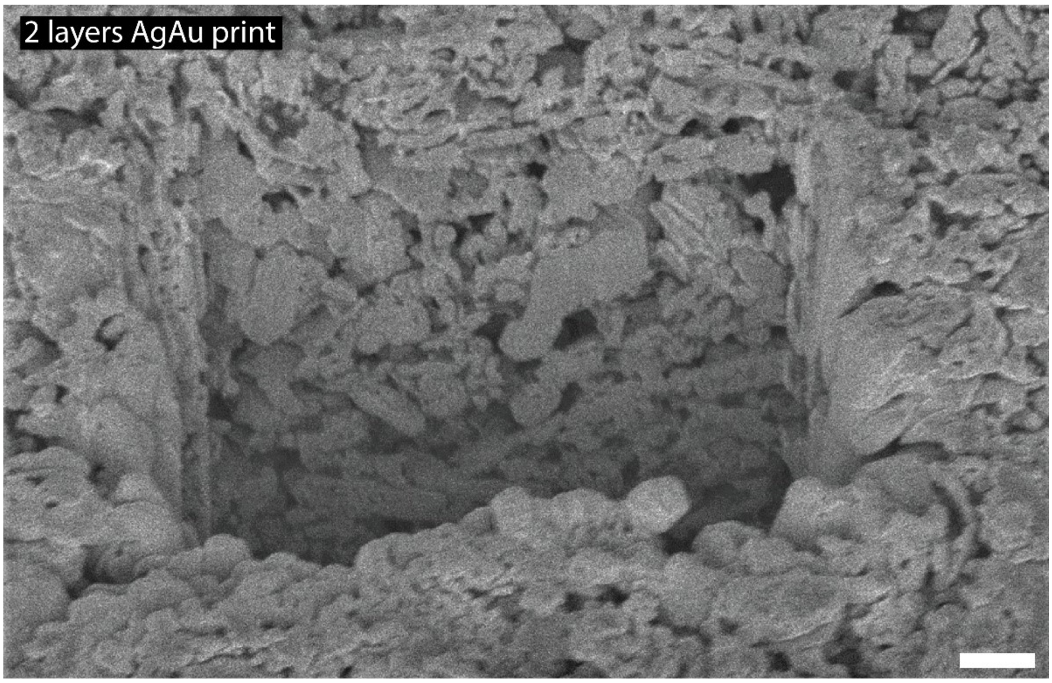

Figure S16. FIB-SEM imaging of AgAu print cut with focused ion beam. Scale bar is 2 μm.

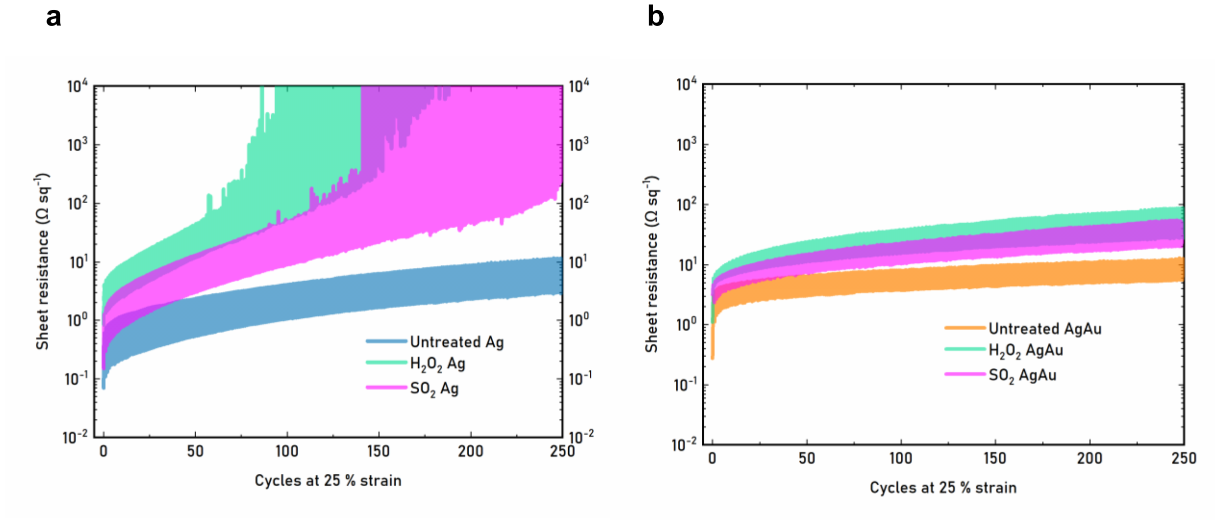

Figure S17. Normalized sheet resistance measured during strain cycling at 25 % max strain of prints made from stretchable (a) Ag ink and (b) AgAu ink, both graphs comparing untreated prints to prints treated with H2O2 (15 mL of 10 % water solution for 3 h) or SO2 (100 ppm, 4 h).

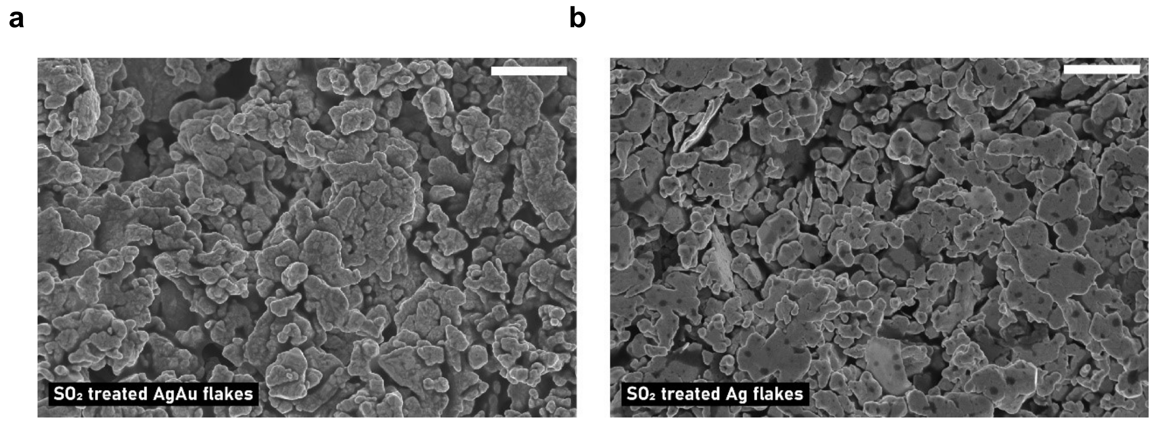

Figure S18. SEM imaging of prints made with ink from (a) AgAu flakes and (b) Ag flakes, both exposed to 100 ppm of a SO2 gas mixture for 4 h. There is no visible difference between these prints and unexposed prints. Scale bars are 2 µm.

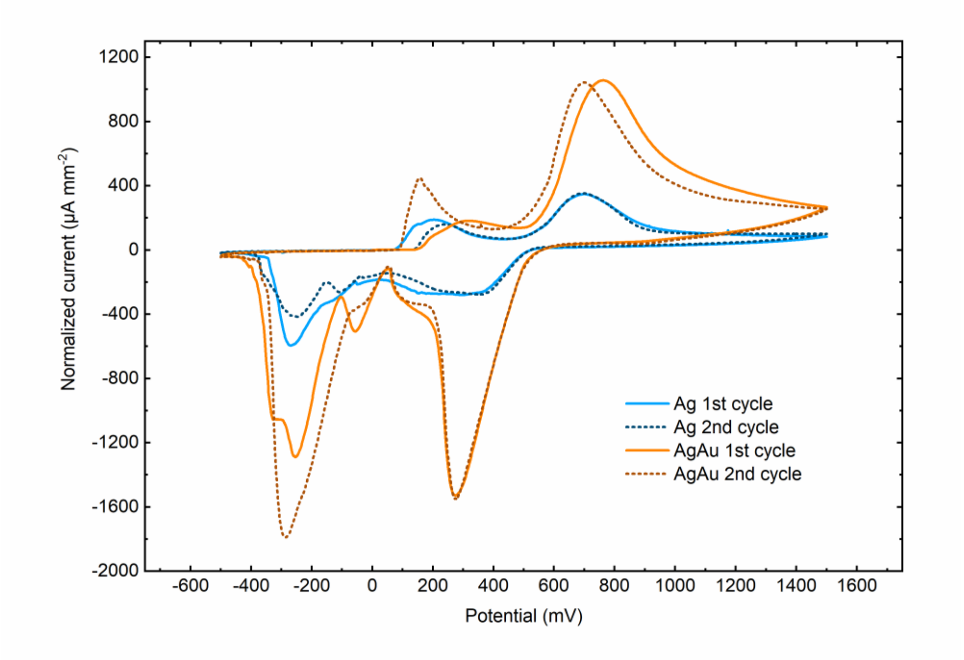

Figure S19. Cyclic voltammetry (scan rate 25 mVs^{-1}) responses obtained for Ag and AgAu printed lines in a 250 mM sulfuric acid solution containing 100 mM NaCl. Potential vs Ag/AgCl reference electrode (3 M NaCl). The Ag oxidation peak at ~200 mV increases in magnitude between the first and second scans for the AgAu print, indicating that more silver is exposed after electrochemically corroding the Au protective layer at high positive potential.

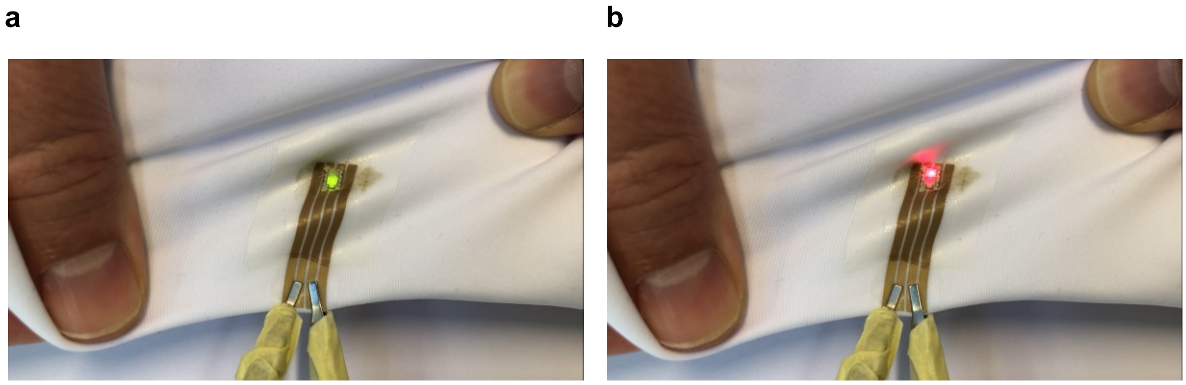

Figure S20. Printed AgAu tracks with mounted LEDs functional during physical manipulation.

a b

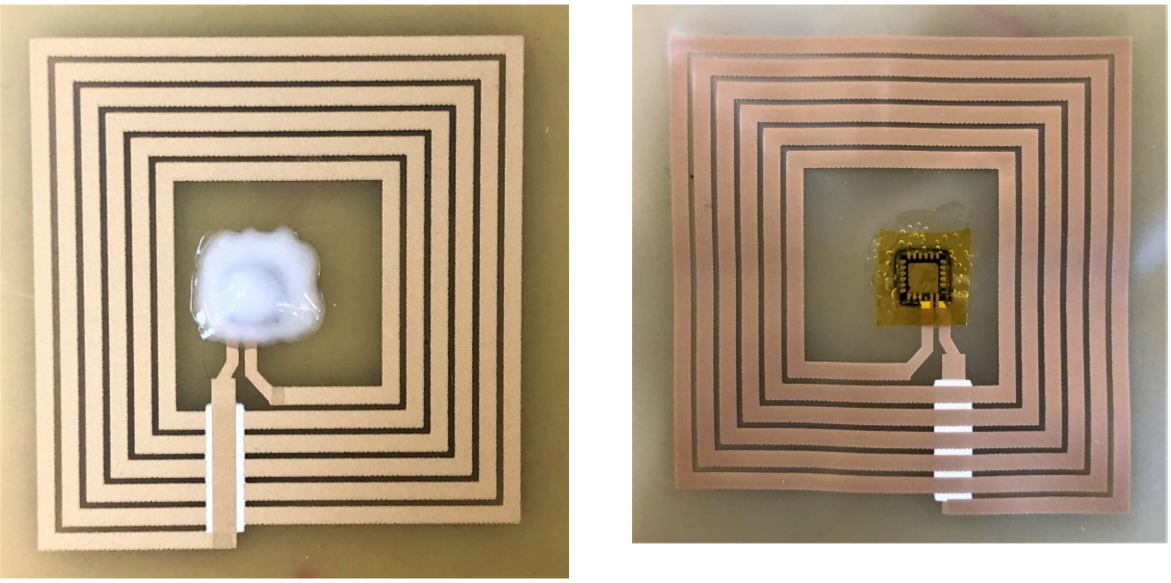

Figure S21. Photo of printed NFC antennas. a) Antenna with mounted microchip and applied PU-based globetop, undried. b) Antenna with mounted microchip, dried globetop, and applied stabilizing kaptop tape on the TPU substrate side (seen from the TPU substrate side).

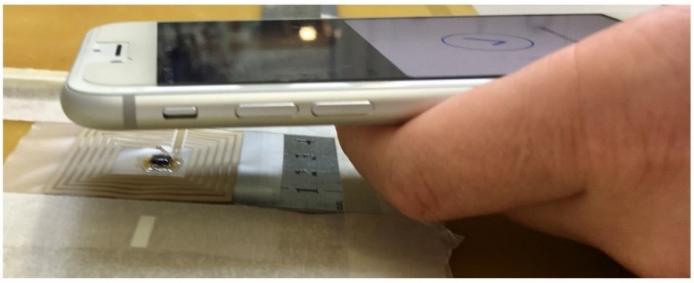

Figure S22. Photo of an iPhone 8 reading the NFC chip in relaxed state.

a b c

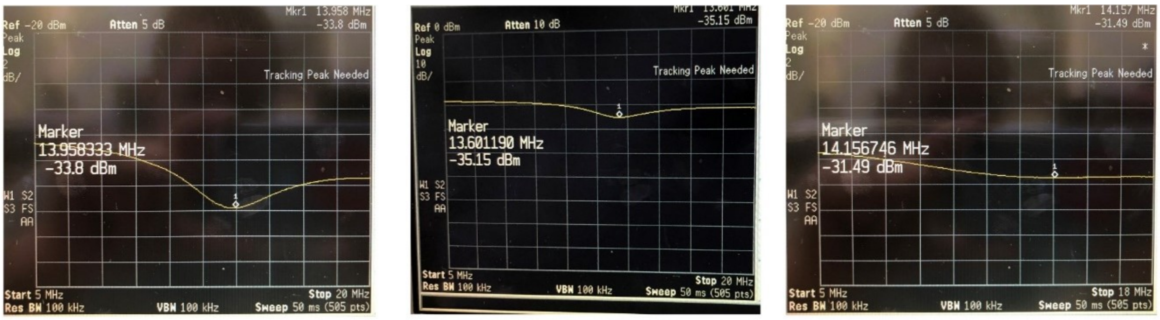

Figure S23. Photos of the output from the measurement setup used to characterize the NFC antennas. a) and b) Example of peaks that yielded valid Q factors. c) Example of a peak where it was no longer possible to deduce a Q factor, nor a distinguished resonance peak.

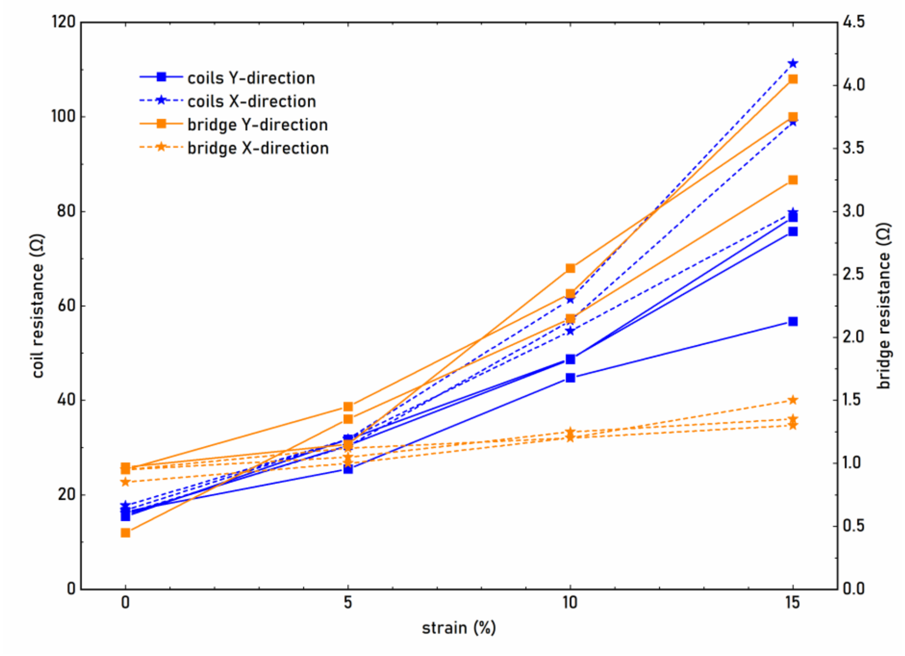

Figure S24. Coil resistance and resistance across the AgAu bridge increasing along with elongation of printed AgAu NFC antennas.
